# Feasibility of Malaria Elimination in Ethiopia

**DOI:** 10.4314/ejhs.v30i4.16

**Published:** 2020-07-01

**Authors:** Gessessew Bugssa, Kiros Tedla

**Affiliations:** 1 Department of Medical Parasitology and Entomology, College of Health Sciences, Mekelle University, Mekelle, Ethiopia

**Keywords:** Malaria, Elimination, feasibility, opportunity, Ethiopia

## Abstract

**Background:**

The problem of malaria is very severe in Ethiopia where it has been the major cause of illness and death for many years. The purpose of this review article is to assess the feasibility of malaria elimination in Ethiopia

**Method:**

To compile this review article, different relevant research articles related to the topic from open access journals were searched using different searching engines such as Google scholar, Science direct, and Pub Med using different key words and phrases

**Result:**

Based on review of the literature, Ethiopia has been trying to control and eliminate malaria for more than 60 years. To assess feasibility of malaria elimination, the WHO assessment tools/recommendations for elimination of malaria were used. Based on WHO parameters, the country has achieved remarkable progress on the fight against malaria during the most recent decades. Malaria morbidity and mortality have been reduced dramatically with intensive use of insecticide residual spray, long lasting insecticide treated nets, chemotherapies, improved diagnosis and case management, improved quality of laboratories, continued support from malaria partners, and political commitment of the Ethiopian government towards malaria prevention and control. Hence, the past achievements and current activities, have led to consider the possibility of malaria elimination in Ethiopia at least by 2030 or beyond

**Conclusion:**

Considering the triumphs achieved so far and the current undertaking efforts, malaria could possibly be eliminated from Ethiopia once and for all

## Introduction

Malaria is a vector-borne disease transmitted mainly through the bites of Anopheles mosquitoes. Five species of the genus *Plasmodium* cause all malarial infections in human beings. Most cases are caused by either *Plasmodium falciparum* or *P. vivax*, but infections can also be caused by *P. ovale*, *P. malariae*, and *P.knowlesi* (1,2). Malaria continues to be a major global health problem, with over 40% of the world's population exposed to varying degrees of malaria risk in some 100 countries (3). Malaria is a major public health problem in Africa with over 200 million cases and nearly one million deaths occurring annually (4).

The problem of malaria is very severe in Ethiopia and has been the major cause of illness and death (4). It is estimated that 75% of the country’s land is malarious with about 68%–70% of the total population living in areas at risk of malaria (4–6). *P.falciparum* and *P.vivax* are the most dominant malaria parasites in Ethiopia accounting for 60% and 40% of malaria cases, respectively (7). The parasites are principally transmitted by the primary mosquito vector known as *Anopheles arabiensis followed by secondary mosquito vectors such as A.pharoensis, A.funestus* and *A.nili* (7,8).

In Ethiopia, there are about 835 districts with different levels of malaria risk with an estimated at-risk population of 50.6 million people (9). In the country, altitude and climate are the most important determinants of malaria transmission (7). The midlands of Ethiopia between 1,000-and 2,200-meters altitude experience seasonal transmission of malaria with sporadic epidemics every 5 to 8 years (8). Transmission is seasonal and largely unstable in character. The major transmission of malaria follows the June-September rains and occurs between September-December while the minor transmission season occurs between April-May following the February-March rains (4).

The disease was one of the leading health problems in Ethiopia with an average of 5 million cases a year (5) and 9.5 million cases per year between 2001 and 2005(10). In 2000, malaria caused around 29000 deaths of children which signified that nearly 80 children died in a day in the country (11). According to a 2005 Ethiopian Demographic Health Survey, malaria was the primary cause of health problems, accounting for 17% of outpatient visits, 15% of hospital admissions, and 29% of in-patient deaths (12). According to Ethiopian Malaria Indicator Survey (EMIS) (13), the prevalence of malaria is estimated to be 1.3%. It is also well evidenced that Ethiopia, one of the Sub-Saharan countries in Africa, shares 6% of the burden (14).

### Purpose of this review

The purpose of this review is to assess the feasibility of malaria elimination from Ethiopia. This review would help to reveal the possibility of malaria elimination from the country to the Ethiopian government, and different stake holders working on malaria so as to enhance their efforts towards the success of malaria elimination.

## Methods

### Search strategy

To compile this review article, different relevant research articles related to the topic from open access journals were searched using different searching engines such as Google scholar, Science Direct, and Pub Med by using different key words and phrases such as ‘malaria’,‘elimination’, ‘feasibility’, ‘opportunity’, ‘Ethiopia’, etc. The review is based on published data, policy briefs, and stake holders’ documents.

## Results

Although malaria control effort in Ethiopia is challenged by insecticide resistance, drug resistance and climatic changes (15), the country has achieved remarkable progress in the fight against malaria during the most recent decades (16). In view of this, we assessed the feasibility of malaria elimination in Ethiopia using the WHO assessment tools/recommendation for elimination of malaria (17).

### Enhancing and optimizing vector control

**Figure 1:**
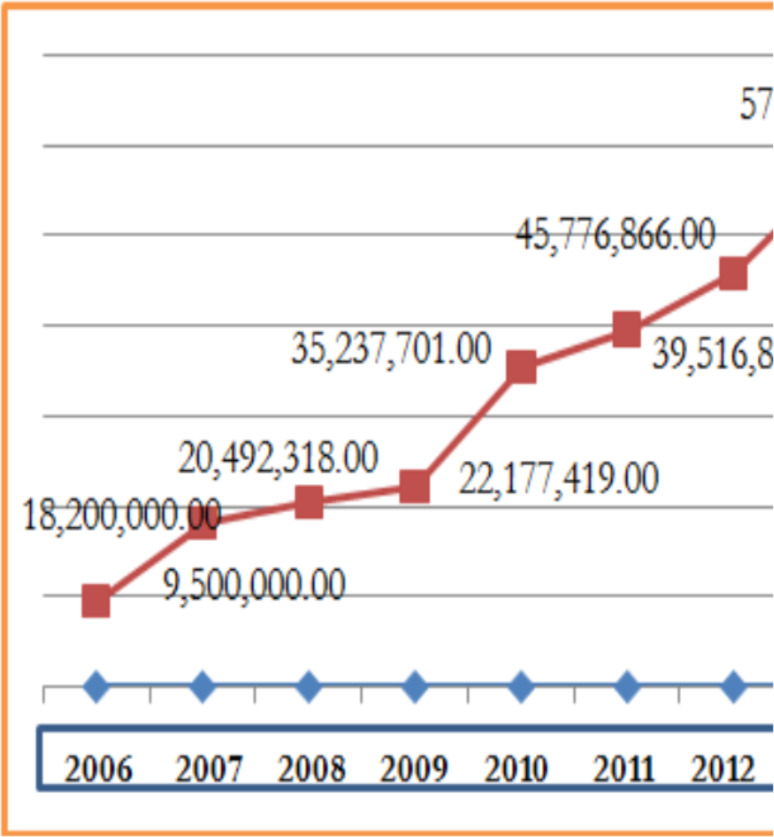
Number of LLINs distributed in Ethiopia, 2006-2013 (modified from FMOH, 2012)

LLINs and IRS are core interventions for reducing the human biting rate and vector survival, which significantly reduce vectorial capacity and transmission (17). In Ethiopia, the use of LLINs and IRS are the mainstay in the prevention and control of malaria (18, 19). Because of the intensive use of these methods in the country in the last decades, sharp declines in the number of malaria cases as well as declines in malaria outbreaks and deaths were observed (16). In children under five years of age, malaria admissions and deaths fell by 81% and 73% respectively between 2001 and 2011. Moreover, malaria related maternal mortality declined by nearly 60%, from 968 maternal deaths per 100,000 live births to 590 between 1990 and 2008. This decrease is correlated with the high distribution of LLINs in the country. By the end of 2007, 20 million LLINs were distributed owing to estimated coverage of almost two LLINs per household in the malaria endemic regions of the country (11,20,21) and more than 57 million nets have been distributed through 2013 (Figure 1)(22).

Moreover, it is also indicated that between 2013 and 2015, a total of 45 million LLINs were distributed throughout the country. The EMIS (2015) showed that 64% of households in malarious areas own at least one LLINs, and 32% of households have at least one for every two people that stayed in the house the night before the survey while 40% of the population slept under LLINs the night before the survey, 45% of children and 44% of pregnant women slept under an LLINs the previous night (23).

**Figure 2:**
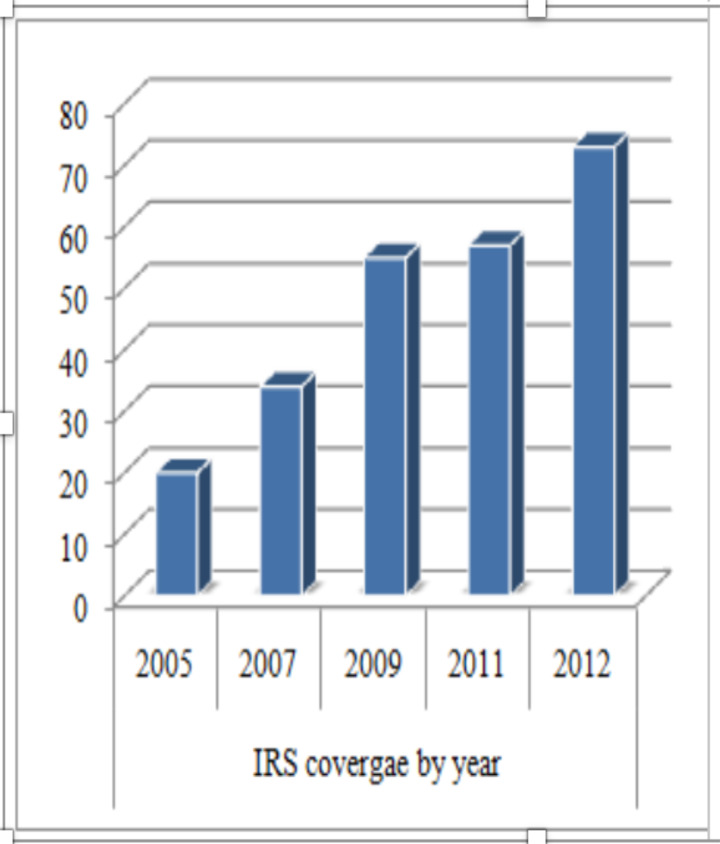
Percentage of households that received IRS from 2005-2012 (Modified from Carter Center, 2013)

The world malaria report (2016) indicated similar report that more than 60% of the Ethiopian population in malarious areas was sleeping under an LLINs or protected with IRS in 2015 (14). It is also evidenced that in 2012, a total of 4383819 (73.1%) of targeted households were sprayed with IRS which was beyond the intended achievements to reach 70% for 2011 (Figure 2) (24).

On top of the above vector control methods, additional or supplementary vector control interventions such as larval source management, community participation and health education also played a great role in minimizing residual transmission of malaria (25).

### Adequate funding and political commitment

An elimination programme will require a short-term as well as a longer-term funding to ensure continued surveillance and control (17). Along with the government of Ethiopia, partners such as the World Bank, the Global Fund, U.K. Department for International Development, UNICEF, and Canadian International Development Agency have been providing financial and material support to fight malaria. One of the strong partners, president malaria initiative (PMI), continues to support malaria prevention and treatment interventions. PMI aimed to reduce malaria morbidity and mortality by 70% by 2015 (26). Partner resources have supported the procurement and free distribution of LLINS, IRS, anti-malarial drugs, as well as the strengthening of the supply chain (27). PMI estimates that at projected funding levels, there will be surpluses of ACT supplies and RDTs for each year from 2015–2017 (28). Other components have included capacity building for other functions, especially procurement and monitoring and evaluation (27). Besides, the Government of Ethiopia planned to commit $66,065,284 or nearly 15% of the total funding requirement towards malaria control and elimination activities for 2015–2017. Ethiopia’s domestic malaria spending has also increased significantly over the past 15 years. Projected 2015 malaria funding of $21,321,519 represents a 247% increase over the 2010 spending level of $6,144,036 and a more than tenfold increase over the 2001 spending level (28).On top of this, strong political commitment is one of the key factors for further progress towards malaria elimination (29). Hence, the presence of adequate funding from the government and/or partners, and strong political commitment are of the good opportunities in realization of malaria elimination (15).

### Enhancing/optimizing case detection and management

It is evident that accurate diagnosis remains essential to target antimalarial drugs, reduce transmission and enable management of cases. A declining level of transmission demands new diagnostic strategies and active case detection (30). In the past decade, Ethiopia has rapidly expanded health institutions, such as district hospitals, health centers and health posts, and deployed a large number of health professionals in all parts of the country (15). The number of health facilities nationally has progressively increased from 3612 in 2000 to 6604 in 2005, and the total number of health facilities in 2016 was 20283 (31). As a result, the country has achieved remarkable progress in the fight against malaria during the most recent decades through strong preventive and case management interventions especially with large engagement of the Health Extension Workers (HEWs) and the Health Development Army volunteers providing community-based care at the household level (15). The existence of HEWs at grass root level in the Ethiopia health system is one of the opportunities to implement a mix of malaria prevention and control tools as they can be involved in malaria case management, identify transmission foci, coordinate IRS and LLINs operation, perform surveillance and carry out information, education and communication to prevent malaria transmission. Moreover, in 2004, the FMoH introduced ACT as the first-line drug for treatment of *P.falciparum* malaria as well as RDTs to improve diagnosis (31). Hence, the introduction of improved diagnostic facilities is providing timely, accurate and reliable results to support diagnosis, outbreak investigations, confirm clinical diagnoses, conduct accurate infectious disease surveillance, and direct public healthcare policy (30). In combination, all these factors facilitate the way to enter the path to pre-elimination/elimination phase.

### Surveillance

Surveillance is recognized as an intervention for malaria eradication in Malaria Eradication Programme. In elimination settings, malaria surveillance comprises a set of responses that should allow (i) detection of all malaria infections (ii) prevention of onward transmission from each case through rapid, radical treatment and vector control; and (iii) identification, investigation, classification and management of all transmission foci with appropriate measures to terminate transmission as soon as possible (17). The Health Extension System in Ethiopia proves a unique opportunity for continuous surveillance throughout the country, because each *kebele* (lowest administrative unit) has a health post staffed by HEWs that routinely test for malaria using RDTs. Besides, health facilities provide longitudinal data on a large number of patients at different spatial and temporal points if documented properly. To this end, the Ethiopian recording system is being improved through the introduction of health management information systems (HMIS) which helps in appropriate reporting and planning in control and prevention of malaria programs (32).

### Quality assurance of laboratories

Quality assurance within national malaria control programmes and national malaria ensures high-quality laboratory diagnosis and treatment of malaria in a country (15). Quality laboratory diagnosis depends on adequate and improved diagnostic facility, which requires adequate physical infrastructure and supplies of materials and reagents, uninterrupted electricity and running water, trained personnel, policy and strategic plan and synergy with clinical and research services. Accurate diagnosis is essential not only to target anti-malarial drugs, but also to enable effective management of other febrile and infectious diseases. Microscopy and RDTs are being used for the diagnosis of malaria in public healthcare facilities of Ethiopia (31).

## Discussion

Global reports indicate that great advances have been made in the past decades in the fight against malaria which resulted in profound reduction of malaria related morbidity and mortality and more than 100 countries in the world freed from malaria (33). The triumphs achieved so far are due to massive scale-up of the vector control interventions using long lasting insecticide-treated bed nets (LLINs) and indoor residual spraying (IRS), as well as introduction of malaria rapid diagnostic tests (RDTs) for better malaria diagnosis and use of highly effective artemisinin-based combination therapies (ACTs) (34). To this end, WHO’s *Global Technical Strategy for Malaria (GTS)*, recently endorsed by the World Health Assembly in 2015, and the Roll Back Malaria (RBM) Partnership’s *Action and Investment to defeat Malaria (AIM)* have embraced the goal of a “world free of malaria” and have put forward ambitious targets of reducing malaria case incidence and mortality rates globally by at least 90% by 2030 with milestones for measuring progress in 2020 and 2025 (35). Different countries are situated at different points along the road to malaria elimination depending on the national health system, the level of investment in malaria control and other biological, environment, social, demographic, political and economic factors (36). As a result, Ethiopia is moving to the malaria pre-elimination phase as many malaria districts have reached pre-elimination levels of transmission qualifying the WHO criteria of being under pre-elimination phase (37). In line with this, Ethiopia has developed a National Malaria Strategic plan (NMSP, 20142020) to end this devastating disease (38). In the past, multiple NMSPs have been made in Ethiopia to control malaria over the last decade: 2001–2005, 2006–2010, and 2010–2013. The current NMSP (2014–2020), however, focuses on transitioning from malaria control to malaria pre-elimination/elimination in Ethiopia (9, 39). Hence, Ethiopia aims near zero malaria deaths, reduced malaria cases by 75% from baseline of 2013, and malaria eliminated in selected areas by the year 2020 and beyond (38). However, there are arguments on the realization of malaria elimination from Ethiopia. Consequently, it is important to discuss past experiences and achievements of the country that have been made and existing opportunities to take a position on the feasibility of malaria elimination from Ethiopia.

It is evident that Ethiopia has been trying to control and eliminate malaria for more than 60 years. The Malaria Eradication Service was established in 1959 making Ethiopia one of the pioneering countries in Africa to embark on a malaria eradication effort. In 2000, the country became a cosignatory to the Abuja Declaration, committing itself to the declaration’s aims of increasing coverage of malaria interventions and reducing malaria mortality by half by 2010 (27). To this end, the Ethiopian government has implemented a centralized approach to bringing the disease under control with significant success. In 2009, after analysis of the results of the 2007 MIS as well as the discussions and recommendations that followed a consultative meeting held in Ethiopia, Ethiopia developed a six-year (2010–15) National Strategic Plan for Malaria Prevention, Control, and Elimination. In view of this strategic plan, top priorities among malaria control strategies were given to community empowerment and social mobilization. These priorities were based on the 2007 MIS results which showed substantial differences between the coverage and utilization of key malaria interventions by the population at risk of malaria. Malaria diagnosis, case management, disease surveillance, and epidemic control were all geared to serve Ethiopia’s goal of shrinking malaria-endemic areas by 2015 and eliminating the disease throughout the country by 2020 (40).

In summary, it can be witnessed that substantial progress has been made in the last decades in controlling malaria in the country through large scale implementation of effective malaria interventions. The magnitude of this progress has led to consider the possibility of malaria elimination in Ethiopia at least by 2030 or beyond. Currently, the country is moving to the malaria pre-elimination phase as many malaria districts have reduced annual malaria incidence exceeding WHO criteria of being under pre-elimination phase (41). Hence, considering the triumphs achieved so far and the current undertaking efforts, malaria could possibly be eliminated from Ethiopia once and for all.

## References

[1] White Nicholas, Sasithon P, Hien Tran (2014). Malaria, seminar. Lancet.

[2] WHO (2016a). Malaria vaccine: WHO position paper. Weekly epidemiological record.

[3] Noppadon T, Chatnapa D, Polrat W, Srivicha K (2009). Malaria Diagnosis: A Brief Review. Korean Journal of Parasitology.

[4] Yeshiwondim A, Sucharita G, Afework T, Dereje O, Patel Hrishikesh (2009). Spatial analysis of malaria incidence at the village level in areas with unstable transmission in Ethiopia. International Journal of Health Geographics.

[5] Senay Gabriel, Verdin James (2005). Developing a Malaria Earky Warning System for Ethiopia. National Center for EROS. Twenty Fifths Annual ESRI International User Conference.

[6] Ayele D, Zewotir T, Mwambi G (2012). Prevalence and risk factors of malaria in Ethiopia. Malaria Journal.

[7] WHO (2017). Malaria. WHO African Region: Ethiopia. http://who.int/countries/eth/areas/cds/malaria/en/.

[8] Alelign A, Dejene T (2016). Current Status of Malaria in Ethiopia: Evaluation of the Burden,Factors for Transmission and Prevention Methods. Acta Parasitologica Globalis.

[9] (2016). President’s Malaria Initiative Ethiopia (PMI). Malaria Operational Plan.

[10] (2008). President’s Malaria Initiative (PMI). Malaria Operational Plan Ethiopia.

[11] Aynalem Adugna Malaria in Ethiopia. http://www.ethiodemographyandhealth.org/MedVectoredDiseasesMalaria.pdf.

[12] Central Statistical Agency (CSA) Ethiopia, and ORC Macro Ethiopia Demographic and Health Survey 2005.

[13] (2011). Ethiopia Malaria Indicator survey (EMIS).

[14] (2016). World Malaria Report (WMR). http://www.who.int/malaria/publications/worldmalariareport2016/report/en/.

[15] Gari Taye, Lindtjørn Bernt (2018). Reshaping the vector control strategy for malaria elimination in Ethiopia in the context of current evidence and new tools: opportunities and challenges. Malar J.

[16] (2009). Global Fund to Fight AIDs, Tuberculosis and Malaria. Early Evidence of Sustainable Impact on Malaria. http://www.theglobalfund.org/documents/publications/onepagers-/Malaria.pdf.

[17] WHO (2017). Global Malaria Programme; A framework for malaria elimination.

[18] Balkew Meshesha, Getachew Alemayehu, Chibsa Shelleme (2012). Insecticide resistance: a challenge to malaria vector control in Ethiopia. Malaria Journal.

[19] Deressa Wakgari, Loha Eskindir, Balkew Meshesha (2016). Combining long-lasting insecticidal nets and indoor residual spraying for malaria prevention in Ethiopia: study protocol for a cluster randomized controlled trial. Trials.

[20] Chibsa Sheleme (2007). Malaria Vector Control Efforts and Challenges in Ethiopia. 4th WIN meeting, Basel, Switzerland.

[21] WHO (2009). Malaria rapid diagnostic test performance.

[22] EthiopiaFederal Ministry of Health (FMOH) (2013). Malaria. http://www.moh.gov.et/malaria.

[23] Ethiopia National Malaria Indicator Survey 2015. https://www.ephi.gov.et/images/pictures/download2009/MIS-2015-Final-ReportDecember-_2016.pdf.

[24] The Carter Center (2013). Summary Proceedings 4th Annual Malaria Control Program Review Ethiopia and Nigeria.

[25] Yohannes M, Haile M, Ghebreyesus TA, Witten KH (2005). Can source reduction of mosquito larval habitat reduce malaria transmission in Tigray, Ethiopia?. Tropical Medicine and International Health.

[26] USAID (United States Agency for International Development) (2019). U.S. President’s Malaria initiative. Malaria, Ethiopia. https://www.usaid.gov/ethiopia/malaria.

[27] Pierre-Louis Anne-Maryse, Qamruddin Jumana, Espinosa Isabel, Challa Shilpa (2011). The Malaria Control Success Story.

[28] PATH, MACEPA (2015). Ethiopia Malaria Financial Landscape; Malaria Funding in Ethiopia – AT a glance. http://www.makingmalariahistory.org/wp-content/uploads/2016/01/EthiopiaFinancial-Landscape-2015.pdf.

[29] World Health Organization (2015). Global Technical Strategy for Malaria 2016–2030.

[30] Animut Abebe, Lindtjørn Bernt (2018). Use of epidemiological and entomological tools in the control and elimination of malaria in Ethiopia. Malaria Journal.

[31] Taffese Hiwot S, Hemming-Schroeder Elizabeth, Koepfli Cristian (2018). Malaria epidemiology and interventions in Ethiopia from 2001 to 2016. Infectious Diseases of Poverty.

[32] Joshua O, Jessica B, Melody M (2014). A description of malaria sentinel surveillance: a case study in Oromia Regional State, Ethiopia. Malaria Journal.

[33] (2013). World Malaria Report. http://www.who.int/malaria/publications/worldmalariareport2013/report/en/.

[34] WHO, World Malaria Report. WHO fact sheet (2015a).

[35] Roll Back Malaria Partnership. Action and Investment to defeat Malaria (AIM). http://www.rollbackmalaria.org/about/about-rbm/aim-2016-2030.

[36] WHO (2016b). Eliminating Malaria. http://apps.who.int/iris/bitstream/handle/10665/205565/WHO_HTM_GMP_2016.3eng.pdf.

[37] Taffese Hiwot S, HemmingSchroeder Elizabeth, Koepfli Cristian, Tesfaye Gezahegn, Lee Ming-chieh, Kazura James (2018). Malaria epidemiology and interventions in Ethiopia from 2001 to 2016. Infectious diseases of poverty.

[38] Ethiopian Federal Ministry of Health (FMOH) National Strategic Plan 20142020.

[39] MACEPA/PATH (2015). Ethiopia: Accelerating Toward Malaria Elimination;Stakeholder Perspectives.

[40] USAID (United States Agency for International Development) (2010). PMI (President’s Malaria Initiative).

[41] President’s Malaria Initiative Ethiopia (PMI) Malaria Operational Plan Fy 2018. https://www.pmi.gov/docs/defaultsource/default-document-library/malariaoperational-plans/fy-2018/fy-2018-ethiopiamalaria-operational-plan.pdf.

